# Association of Prenatal Exposure to Ambient Air Pollution With Circulating Histone Levels in Maternal Cord Blood

**DOI:** 10.1001/jamanetworkopen.2020.5156

**Published:** 2020-05-18

**Authors:** Karen Vrijens, Ann-Julie Trippas, Wouter Lefebvre, Charlotte Vanpoucke, Joris Penders, Bram G. Janssen, Tim S. Nawrot

**Affiliations:** 1 Center for Environmental Sciences, Hasselt University, Diepenbeek, Belgium; 2 Flemish Institute for Technological Research (VITO), Mol, Belgium; 3 Belgian Interregional Environment Agency (IRCELINE), Brussels, Belgium; 4 Hospital East Limburg, Genk, Belgium; 5 Department of Public Health, Environment and Health Unit, Leuven University (KU Leuven), Leuven, Belgium

## Abstract

**Importance:**

Exposure to ambient air pollution has been associated with the risk of carcinogenesis in later life. Changes in histone modifications might have long-term adverse health effects.

**Objective:**

To investigate the association of prenatal exposure to ambient air pollution with levels of circulating total histone H3 and specific trimethylation marks (ie, H3 lysine 4, H3 lysine 36) in maternal cord blood.

**Design, Setting, and Participants:**

The Environmental Influence on Aging (ENVIR*ON*AGE) birth cohort study included 609 mothers and their newborns. Participants were recruited when mothers entered the Hospital East Limburg (Genk, Belgium) for delivery between February 2010 and January 2017. The inclusion criteria were singleton pregnancies and the ability to fill out questionnaires in Dutch. Data analysis was conducted from March to August 2019.

**Exposures:**

Exposure to particulate matter with a diameter less than 2.5 μm (PM_2.5_), black carbon, and nitrogen dioxide during pregnancy was modeled with a high-resolution air pollution model on the basis of maternal address for each trimester of pregnancy as well as for the entire pregnancy.

**Main Outcomes and Measures:**

Circulating total histone H3 levels and specific trimethylation marks (ie, trimethylated H3 lysine 4 and trimethylated H3 lysine 36) in cord blood.

**Results:**

A total of 609 mother-newborn pairs were included in the study. Mean (SD) maternal age was 29.3 (4.6) years, 391 mothers (64.2%) never smoked, and 314 (51.3%) had a high education level. Overall, 322 newborns (52.4%) were boys, and mean (SD) birth weight was 3414 (485) g. Participants experienced mean (SD) exposure to PM_2.5_, black carbon, and nitrogen dioxide of 13.4 (2.6) μg/m^3^, 1.29 (0.31) μg/m^3^, and 17.98 (4.57) μg/m^3^, respectively, during their entire pregnancies. Trimethylated H3 lysine 4 and total histone H3 were positively associated with gestational PM_2.5_ exposure, with a 74.4% increment (95% CI, 26.7% to 140.2%, *P* < .001) and a 40.2% increment (95% CI, 24.1% to 58.3%, *P* < .001), respectively, observed for each 5-μg/m^3^ increase in PM_2.5_ exposure during the entire pregnancy. For the same exposure window, trimethylated H3 lysine 36 levels were inversely associated with PM_2.5_ exposure (−34.4%; 95% CI, −50.1% to −13.7%; *P* = .003). Exposure to black carbon during the entire pregnancy was positively associated with trimethylated H3 lysine 4 (38.4%; 95% CI, 6.2% to 80.3%; *P* = .003).

**Conclusions and Relevance:**

Associations of ambient air pollution with cord plasma histone H3 modifications during early life might indicate that circulating histones are a risk factor in the development of air pollution–associated disease later in life. Additional study is required to correctly estimate the long-term consequences of our findings.

## Key Points

**Question:**

Is prenatal exposure to ambient air pollution associated with levels of circulating total histone H3 and specific trimethylation marks in cord blood?

**Findings:**

In this cohort study with 609 mother-newborn pairs, prenatal exposure to ambient air pollution was positively associated with circulating total histone H3 levels and with trimethylated histone H3 lysine 4 levels and negatively associated with trimethylated histone H3 lysine 36 levels.

**Meaning:**

The findings of this study suggest that cord plasma histone H3 modifications during early life might indicate circulating histones are a risk factor in the development of air pollution–related disease later in life.

## Teaser

This cohort study investigates the association of prenatal exposure to ambient air pollution with levels of circulating total histone and specific trimethylation marks in maternal cord blood.

## Introduction

The fetus is particularly vulnerable to adverse effects of environmental exposures, given that it is growing and developing at a rapid pace.^1^ Epidemiologic studies have shown that exposure to airborne particulate matter (PM) during gestation increases the risk of low birth weight and preterm birth.^2^

The developmental origin of health and disease concept explains how challenges early in life can affect lifelong health.^3^ Determining which molecular mechanisms underlie prenatal exposure to air pollution is important for the discovery of potential novel biomarkers of exposure as well as for the identification of potential mediators between exposure and disease.

Air pollution is known to, at least in part, exert its effects on human health by upregulating oxidative stress and pro-inflammatory responses.^4^ Epigenetic mechanisms are involved in the regulation of oxidative stress as well as in inflammatory responses.^5^ Histones are small, abundant proteins that help form nucleosomes. Circulating histones can either be secreted from living or apoptotic cells. When released in circulation, they mediate the inflammatory response and can lead to endothelial dysfunction and organ failure.^6^

Histone posttranslational modifications (PTMs) are an epigenetic trait induced by oxidative stress^7^ and specific inflammatory mediators.^8^ Posttranslational modifications have a critical role in the regulation of nucleosome dynamics and the processes of DNA transcription, replication, and repair.^9^ Histones have also been shown to have toxic and pro-inflammatory activities when they are released into the extracellular space.^6^

We hypothesized that histone modifications may serve as a molecular pathway in the response to prenatal ambient air pollution exposure. Modifications to histone H3 are the most-studied histone changes at present, and these changes have been associated with changes in gene expression.^10^ Previous evidence has identified specific histone H3 modifications after exposure to organic chemical compounds,^11^ heavy metals,^12,13,14^ and, recently, traffic-related PM exposure in adults.^15^ To our knowledge, no human studies have reported on prenatal air pollution exposure and its role in histone H3 modifications.

Trimethylated H3 lysine 4 (H3K4me3) is a modification typically associated with transcriptional activity. We selected H3K4me3 for this study because it has been shown to play an important role in memory and cognitive impairment. Because air pollution exposure is known to affect the brain but the underlying molecular mechanisms are poorly understood, we hypothesized that HK4me3 might play an important function here. Global levels of H3K4me3 are increased in the hippocampus during memory formation, and H3K4 methyltransferases and H3K4 demethylases have been associated with impaired cognition in neurologic disorders.^16^

Methylation of H3 lysine 36 (H3K36) plays crucial roles in the regulation of a wide range of biological processes. Deregulation of H3K36 methylation is associated with disease, including cancer. This has been shown for lung cancer, in which alterations in histone lysine methylation are associated with clinical prognosis.^17^ The H3K36 demethylase KDM2A has been shown to be frequently upregulated in non–small cell lung cancer tumors and to promote tumor growth and invasiveness.^18^

In this study, we measured 2 specific histone H3 modifications (ie, H3K4me3 and H3K36me3) as well as total histone H3 levels in cord blood samples collected from newborns from the Environmental Influence on Aging (ENVIR*ON*AGE) birth cohort. To our knowledge, this is the first report studying gestational air pollution exposure and circulating levels of histones and histone modifications. We selected these PTMs as a discovery study on the potential role of histone PTMs in the adverse effects of gestational exposure to ambient air pollution.

## Methods

### Study Design and Population

From the ongoing population-based birth cohort study ENVIR*ON*AGE, 609 mother-child pairs were recruited between February 2010 and January 2017 at Hospital East Limburg (Genk, Belgium) and included in the current study. Women were recruited when they arrived at the hospital for delivery and filled out study questionnaires at the maternity ward before leaving the hospital. Inclusion criteria were singleton pregnancy and the ability to fill out questionnaires in Dutch. The overall participation rate of eligible mothers was 61.0% (1080 of 1770), and we previously demonstrated that the cohort represents births in Flanders well.^19^

Study approval was obtained from the ethics committees of Hospital East Limburg and Hasselt University and has been carried out according to the Declaration of Helsinki.^20^ Written informed consent was obtained from the mothers before participation. This study followed the Strengthening the Reporting of Observational Studies in Epidemiology (STROBE) reporting guideline. Data analysis was conducted from March to August 2019.

Information on maternal age, smoking behavior (mother and coresidents), ethnicity, prepregnancy body mass index (BMI, calculated as weight in kilograms divided by height in meters squared), and parity were obtained through questionnaires. Ethnicity was classified based on the native country of the neonates’ grandparents as either European (≥2 grandparents were European) or non-European (≥3 grandparents were not European). The study questionnaire also asked mothers to provide their residential address and to indicate whether they moved during pregnancy. If they had, they were asked to provide their former residential address. Furthermore, they provided their exact moving date to allow for accurate exposure calculation. Perinatal parameters, such as newborn sex, birth date, birth weight, and gestational age, were collected from birth records. Information on maternal education was missing for 7 individuals, resulting in a final study population of 609 participants.

### Sample Collection

Umbilical cord blood was collected directly after delivery in BD Vacutainer plastic whole blood tubes with spray-coated K2EDTA (BD). Within 20 minutes of cord blood collection, samples were centrifuged at 3200 rpm for 15 minutes to separate plasma from blood. Plasma was collected and stored in Eppendorf tubes at −80 °C until analysis.

### Exposure Assessment

Based on the mother’s residential address, daily mean concentrations of PM with a diameter less than 2.5 μm (PM_2.5_), nitrogen dioxide (NO_2_), and black carbon (BC) in micrograms per cubic meter were estimated using a high-resolution spatial-temporal interpolation method (ie, kriging)^21^ in combination with a dispersion model.^22,23^ This interpolation method uses hourly measured PM_2.5_ pollution data collected at the official fixed-site monitoring stations (34 sites for PM_2.5_, 44 for NO_2_, and 14 for BC) and land-cover data obtained from satellite images.^24^ The model chain provides daily PM_2.5_, NO_2_, and BC values on a dense, irregular receptor grid by using data both from the Belgian telemetric air-quality network and emissions from point sources and line sources. In the Flemish region of Belgium, more than 80% (*R*^2^ = 0.8) of the temporal and spatial variability was explained by this interpolation tool.

The accuracy of the model has also been demonstrated by showing that modeled PM_2.5_ and BC at residence correlates with internal exposure to nano-sized BC particles measured in urine.^25^ Based on daily residential air pollution levels, we averaged trimester-specific exposure during pregnancy (first trimester, date of conception until 13th week; second trimester, 14th until 26th week; and third trimester, 27th week until delivery) and exposure during the entire period of pregnancy (date of conception until delivery). When participants moved during pregnancy, we accounted for these changes in our exposure calculations. The mean daily outdoor temperature (°C) was provided by the Belgian Royal Meteorological Institute.

### Analysis of Total and Modified Histone H3 Levels

EpiQuik Global Tri-Methyl Histone Quantification Kits (Epigentek) were used to analyze and measure H3K4me3 (cat No. 3112), H3K36me3 (cat No.3130), and total histone H3 (cat No. 3091) levels in circulation. Extracellular nucleosomes bearing those modifications were captured in the strip-wells coated with corresponding antibodies. The captured histones were then detected with a labeled detection antibody, followed by a color development reagent. The ratio of methylation is proportional to the intensity of absorbance. We applied 30 μL plasma with 5 control samples added in duplicate with the same volume. The 5 control samples included on each plate were used to calculate a plate-level normalization factor to minimize plate-to-plate variability. The measurements were done according to the instructions of the manufacturer, and absorbance was measured at 450 nm on a FLUOstar Omega microplate reader (BMG Labtech). To minimize interassay variability, the 609 samples were randomly distributed across nine 96-well plates, including 5 control samples on all plates. In a pretesting phase, we determined the intra- and interassay variation of histone methylation assays. The intra-assay coefficients of variation were 7.2%, 6.3%, and 6.4% for H3K4me3, H3K36me3, and total histone H3, respectively. The interassy coefficients of variation were 18.4%, 11.8%, and 9.0% for H3K4me3, H3K36me3, and total histone H3, respectively. To accurately determine levels of trimethylated and total histone H3 levels, a standard curve using the control delivered with the kit was included on each plate, and the slope of this calibration curve was used in following calculation: H3K4me3 (ng/ml) = (sample optical density − blank optical density) / ([slope × 30 μL] × 1000).

### Statistical Analysis

We used SAS software version 9.4 (SAS Institute) for statistical analysis. The quantities of H3K4me3, H3K36me3, and total histone H3 were log-transformed because of their nonnormal distribution. The collected data are presented as categorical data with numbers and percentages and as continuous data with means and SDs. For histone methylation levels, geometric means and 25th and 75th percentiles are given. The association of levels of total histone H3 in cord blood with prenatal air pollution exposure was assessed using a linear regression model, while accounting for the following covariates: newborn sex, ethnicity (European or non-European), gestational age (in weeks), season of delivery (winter, spring, summer, or autumn), trimester-specific apparent temperature, maternal age (in years), smoking status (never smoked, formerly smoked, or currently smokes), educational status (coded as low, no diploma or primary school; middle, high school; and high, college or university degree), prepregnancy BMI, and parity (1, 2 or ≥3). In a sensitivity analysis, we additionally adjusted for white blood cell count and the percentage of neutrophils. As data on blood cell count was missing for 116 individuals, the sensitivity analysis was performed with the remaining 493 individuals. Statistical significance was set at *P* < .05, and all tests were 2-tailed.

## Results

### General Characteristics of the Study Population

Detailed maternal and newborn characteristics are shown in Table 1. The mean (SD) maternal age was 29.3 (4.6) years, 391 mothers (64.2%) never smoked, and 314 (51.3%) had a high educational level. The mean (SD) maternal prepregnancy BMI was 24.3 (4.6). Overall, 322 newborns (52.4%) were boys, and 536 (88.0%) had European descent. The mean (SD) gestational age was 39 (1.7) weeks (range, 36-41 weeks). Mean (SD) birth weight was 3414 (485) g. Residential prenatal air pollution exposure is summarized in Table 2. Mean (SD) PM_2.5_, BC, and NO_2_ exposure was 13.4 (2.6) μg/m^3^, 1.29 (0.31) μg/m^3^, and 17.98 (4.57) μg/m^3^, respectively, during the entire pregnancy.

**Table 1.** Demographic Characteristics of the Study Population^a^

Characteristic	No. (%) (N = 609)
Mother	
Age, mean (SD), y	29.3 (4.6)
Pregestational body mass index, mean (SD)^b^	24.6 (4.6)
Educational level	
Low	75 (12.4)
Middle	220 (36.3)
High	314 (51.3)
Parity	
1	336 (55.5)
2	202 (33.0)
≥3	71 (11.5)
Smoking status	
Never	391 (64.2)
Before pregnancy	152 (25.0)
During pregnancy	66 (10.5)
Newborn	
Boy	322 (52.4)
Race/ethnicity	
European	536 (88.0)
Non-European	73 (12.0)
Gestational age, mean (SD), wk	39 (1.7)
Birth weight, mean (SD), g	3414 (485)
Season of birth	
Winter	156 (25.8)
Spring	157 (25.8)
Summer	144 (23.4)
Autumn	152 (25.0)
White blood cell count, mean (SD), /μl	15200 (4700)
Neutrophils, mean (SD), %	53.2 (9.4)
H3K4me3, log, median (IQR), ng/mL	10.3 (6.3-23.6)
H3K36, log, median (IQR), ng/mL	8.9 (4.6-16.6)
Total histone H3, log, median (IQR), ng/mL	32.3 (27.3-37.9)

Abbreviations: IQR, interquartile range; H3K4me3, trimethylated H3 lysine 4; H3K36, H3 lysine 36.

SI conversion factor: To convert neutrophils to proportion of 1.0, multiply by 0.01; white blood cell count to ×10^9^/L, multiply by 0.001.

^a^ Means are presented as geometric means (ie, 25th to 75th percentile).

^b^ Body mass index calculated as weight in kilograms divided by height in meters squared.

**Table 2.** Characteristics of Air Pollution Exposure Data for the Study Population

Exposure	Period	Mean (SD) [IQR]
PM_2.5,_ μg/m^3^	Trimester 1	13.30 (4.64) [6.91-19.69]
	Trimester 2	13.52 (4.72) [6.12-20.92]
	Trimester 3	13.50 (5.15) [5.99-21.01]
	Entire pregnancy	13.43 (2.55) [9.72-17.14]
BC, μg/m^3^	Trimester 1	1.30 (0.38) [0.76-1.84]
	Trimester 2	1.30 (0.44) [0.72-1.88]
	Trimester 3	1.27 (0.42) [0.69-1.85]
	Entire pregnancy	1.29 (0.31) [0.86-1.72]
NO_2,_ μg/m^3^	Trimester 1	17.95 (5.57) [10.56-25.34]
	Trimester 2	18.10 (5.97) [9.58-26.62]
	Trimester 3	17.89 (5.99) [9.61-26.17]
	Entire pregnancy	17.98 (4.57) [11.84-24.12]

Abbreviations: BC, black carbon; IQR, interquartile range; NO_2_, nitrogen dioxide; PM_2.5_, particulate matter with a diameter less than 2.5 μm.

### Association of Air Pollution Exposure With Cord Blood H3K4, H3K36, and Total Histone H3 Levels

#### PM_2.5_ Exposure

**Figure 1.**
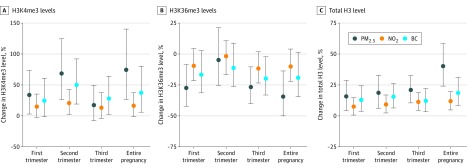
Results of the Main Analysis Estimates are shown with 95% CIs (error bars) for the relative percentage change in histone H3 methylation for a 5-μg/m^3^ increment in exposure for particulate matter with a diameter less than 2.5 μm (PM_2.5_) and nitrogen dioxide (NO_2_), and a 0.5-μg/m^3^ increment in exposure for black carbon (BC). Estimates are from linear regression models and were adjusted for maternal education level, maternal smoking, maternal age, maternal prepregnancy body mass index, parity, newborn sex, gestational age, season of delivery, and trimester-specific apparent temperature. H3K4me3 indicates trimethylated H3 lysine 4; H3K36me3, trimethylated H3 lysine 36.

Cord blood H3K4me3 levels were positively associated with in utero PM_2.5_ exposure during the first trimester (33.9%; 95% CI, 3.1% to 73.4%; *P* = .04), second trimester (68.5%; 95% CI, 26.4% to 124.5%; *P* = .01), and the entire pregnancy (74.4%; 95% CI, 26.7% to 140.2%; *P* < .001), and total histone H3 levels were positively associated with in utero PM_2.5_ exposure for each trimester and the entire pregnancy (first trimester: 15.8%; 95% CI, 4.3% to 28.7%; *P* = .03; second trimester: 18.7%; 95% CI, 6.1% to 32.8%; *P* = .005; third trimester: 21.0%; 95% CI, 10.5% to 32.5%; *P* < .001; entire pregnancy: 40.2%; 95% CI, 24.1% to 58.3%; *P* < .001). Cord blood H3K36me3 levels were inversely associated with first trimester (−27.4%; 95% CI, −42.3% to −8.5%; *P* = .04), third trimester (−26.7%; 95% CI, −40.0% to −10.4%; *P* = .009), and entire pregnancy (−34.4%; 95% CI, −50.1% to −13.7%; *P* = .03) PM_2.5_ exposure after correction for maternal education, smoking, age, prepregnancy BMI, and parity, newborn sex, gestational age, season of delivery, and trimester-specific apparent temperature (Figure 1).

#### NO_2_ Exposure

The estimated overall (weeks 1-40) change in H3K4me3 level for a 5-μg/m^3^ increment of NO_2_ exposure was 16.7% (95% CI, −0.8% to 37.4%; *P* = .003), and total histone H3 levels showed a 11.9% increase (95% CI, 4.8% to 20.0%). Trimester-specific estimates were only significant for the second trimester for H3K4me3 (20.8%; 95% CI, 2.4% to 42.5%; *P* = .02), whereas for total histone H3 levels, first trimester NO_2_ exposure was associated with an increase in total histone H3 levels of 7.5% (95% CI, 0.7% to 14.7%; *P* = .04); second trimester, 9.4% (95% CI, 2.4% to 16.9%; *P*=.03); and third trimester, 11.3% (95% CI, 4.3% to 18.7%; *P* = .005).

#### BC Exposure

We found that H3K4me3 levels were significantly associated with second and third trimester as well as entire pregnancy BC exposure (second trimester: 51.4%; 95% CI, 19.2% to 92.1%; *P* = .04; third trimester: 29.9%; 95% CI, 2.9% to 64.2%; *P* = .01; entire pregnancy: 38.4%; 95% CI, 6.2% to 80.3%; *P* = .003), while H3K36me3 levels were significantly and inversely associated only with third trimester exposure (−19.1%; 95% CI, −33.1% to −2.3%; *P* = .02). All investigated time windows were associated with total histone H3 levels in cord blood (first trimester: 13.6%; 95% CI, 3.8% to 24.3%; *P* = .02; second trimester: 15.9%; 95% CI, 6.4% to 26.2%; *P* = .01; third trimester: 12.3%; 95% CI, 3.4% to 22.1%; *P* = .02; entire pregnancy: 19.2%; 95% CI, 8.4% to 31.1%; *P* = .002) (Figure 1).

#### Sensitivity Analysis

**Figure 2.**
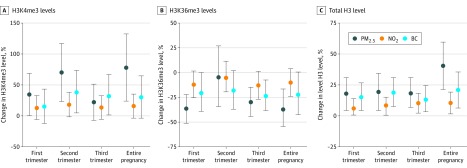
Results of the Sensitivity Analysis Including Percentage of Neutrophils and White Blood Cell Count Estimates are shown with 95% CIs (error bars) for the relative percentage change in histone H3 methylation for a 5-μg/m^3^ increment in exposure for particulate matter with a diameter less than 2.5 μm (PM_2.5_) and nitrogen dioxide (NO_2_), and a 0.5-μg/m^3^ increment in exposure to black carbon (BC). Estimates were adjusted for maternal education level, maternal smoking, maternal age , maternal prepregnancy BMI, parity, newborn sex, gestational age, season of delivery, trimester-specific apparent temperature, white blood cell count, and percentage of neutrophils. H3K4me3 indicates trimethylated H3 lysine 4; H3K36me3, trimethylated H3 lysine 36.

Because we did not know the exact cellular composition of our samples and this might affect our results, we performed an additional analysis in which we corrected for white blood cell counts and percentage of neutrophils in the samples. Although blood cell counts were missing for 116 samples, the characteristics of the population did not change (data not shown). Results were similar to our main analysis (Figure 2).

## Discussion

Epigenetic modifications, such as histone modifications, are involved in mediating how early life environment affects later health. Intracellular histones are known to play an important role in the regulation of transcription and gene expression,^26^ whereas extracellular histones play an important role in inflammation. Extracellular histones induce apoptosis in several human cell types.^27,28^ It has been demonstrated that severely damaged tissues release high amounts of nucleosomes and free histones into the bloodstream, aggravating the clinical features of a trauma. Circulating histones act as mediators for distant organ damage (ie, lungs) through interaction with membrane phospholipids, causing cellular calcium influx.^26^ Histones released in the bloodstream were found in patients with severe blunt trauma, pancreatitis, and sepsis.^6^ Based on their pathological properties, extracellular nucleosomes and histones are considered pathogen-associated molecular patterns and damage-associated molecular patterns. These molecules are able to activate the innate immune system through toll-like receptors.^29^ Once activated by toll-like receptors, endothelial cells were found to secrete mediators including cytokines, chemokines, reactive oxygen species, and nitric oxide.^30^

The most important intracellular function of histones is to construct nucleosomes, which form the basic structure of chromatin. Changes in the structure and function of chromatin affect gene transcription and expression through fine-tuned mechanisms. Covalent modifications of the N-terminal tail of core histones are important regulators of chromatin status, structure, and gene expression. Methylation of histone lysine can either silence or activate gene transcription, depending on the target residue.^31^ Histone modification levels can predict levels of gene expression, and actively transcribed genes are characterized by high levels of H3K4me3 in the promoter region.^32^

We do not know whether the cord blood circulating histones we measured are freely circulating or encapsulated in extracellular vesicles, although it is known that extracellular vesicles can capsulate histones and nucleosomes.^33^ In future research, it would be interesting to extract extracellular vesicles from the cord blood plasma directly and determine whether the histones are indeed found in circulating in extracellular vesicles or rather free circulating.

To our knowledge, this is the first report investigating the association of circulating trimethylated and total histone H3 levels early in life with gestational particulate matter exposure. Our findings showed a positive association between air pollution exposure and total histone H3 levels. This is in accordance with the observation that levels of circulating histones are increased in inflammation and sepsis^6^ and that circulating histone levels are associated with acute lung injury after trauma. In mice, increased levels of circulating histones lead to an increase in the size and severity of stroke.^26^ Our results are in agreement with cohort studies on environmental exposure measured in peripheral blood samples, including arsenite^34^ and nickel.^35^ Although it is known that H3K4me3 is associated with DNA transcription activation, the exact mechanism behind its association with environmental exposure is poorly understood. The inverse association we observed between H3K36me3 and pollution exposure corresponds to results from a study among truck drivers in Beijing, in which 14-day average ambient PM with a diameter less than 10 μm exposure was significantly and inversely associated with H3K36me3 levels in plasma leukocytes from adults.^15^ It appears that both short- and long-term air pollution exposure affect H3 methylation levels, given that the study by Zheng et al^15^ considered 14-day moving averages for PM exposure, whereas we observed significant associations between trimester and entire pregnancy exposure and H3 trimethylation marks. All the previously mentioned studies have measured cellular levels of histones and histone modifications, whereas our study is the first, to our knowledge, to report circulating histone levels in association with air pollution exposure.

The public health significance of our findings is currently not known. Air pollution exposure is known to affect child health, considering that it has been associated with a decrease in birth weight^36^ and childhood blood pressure.^37^ However, insults to the genome in the perinatal period can contribute to carcinogenesis during the life course and may be more important relative to other life stages because of the higher probability that mutated and genomically unstable cells could populate the rapidly growing tissues of an infant.^38,39^ Exposure to traffic-related air pollution was shown to increase the risk of pediatric acute leukemia in the Italian Studio Epidemiologico sui Tumori Infantili Linfoemopoietici (SETIL) study.^40^ Therefore, in utero exposures affecting histone (methyl) levels are potentially important in creating an adverse environment that enables carcinogenesis in later life.

### Limitations

The current findings must be interpreted in the context of their limitations. At this time, we cannot make any predictions on the role of levels of trimethylated and total histone H3 in cord blood in postnatal child health or adult disease risk. Furthermore, as our analyses were performed in plasma extracted from cord blood, we do not know which cell types were responsible for the presence of circulating histones in our samples; further research focusing on which cell types drove our observations is warranted. Furthermore, although our results were consistent after multiple adjustments, we cannot exclude the possibility of residual confounding by some unknown factor that is associated with both cord blood histone H3 levels and air pollution exposure.

A potential source of bias for studies on air pollution exposure is exposure misclassification. Errors in the measurement of PM by monitoring stations and interpolation methods used to estimate individuals’ PM exposure may be a potential source of information bias. Personal PM exposure might be quite different from the estimated outdoor PM exposure since participants may spend a large amount of time indoors and outside the direct environment of their home address, which results in exposure misclassification. Under the assumption that this is not a systematic error (ie, nondifferential misclassification), it would lead to attenuation of effect estimates and not to a greater risk of false-positive results.

## Conclusions

In this cohort study, we identified histone modifications at birth that were associated with prenatal exposure to ambient air pollution. These modifications might have lasting consequences and contribute to how early life environment affects later health.
